# Verification of microRNA expression in human endometrial adenocarcinoma

**DOI:** 10.1186/s12885-016-2296-z

**Published:** 2016-04-02

**Authors:** Sanja Jurcevic, Karin Klinga-Levan, Björn Olsson, Katarina Ejeskär

**Affiliations:** 1 Systems Biology Research Centre – Biomedical genetics, School of Bioscience, University of Skövde, Skövde, Sweden; 2 Systems Biology Research Centre – Bioinformatics, School of Bioscience, University of Skövde, Skövde, Sweden

**Keywords:** Endometrial adenocarcinoma, microRNA, mir-34a, Target genes

## Abstract

**Background:**

MicroRNAs are small non-coding RNAs that have been implicated in tumor initiation and progression. In a previous study we identified 138 miRNAs as differentially expressed in endometrial adenocarcinoma compared to normal tissues. One of these miRNAs was miRNA-34a, which regulates several genes involved in the Notch pathway, which is frequently altered in endometrial cancer. The aims of this study were to verify the differential expression of a subset of miRNAs and to scrutinize the regulatory role of mir-34a on the target genes *NOTCH1* and *DLL1*.

**Methods:**

Twenty-five miRNAs that were previously identified as differentially expressed were subjected to further analysis using qPCR. To investigate the regulation of *NOTCH1* and *DLL1* by mir-34a, we designed gain- and loss-of-function experiments in Ishikawa and HEK293 cell lines by transfection with a synthetic mir-34a mimic and a mir-34a inhibitor.

**Results:**

Of the 25 validated miRNAs, seven were down-regulated and 18 were up-regulated compared to normal endometrium, which was fully consistent with our previous findings. In addition, the up-regulation of mir-34a led to a significant decrease in mRNA levels of *NOTCH1* and *DLL1*, while down-regulation led to a significant increase in mRNA levels of these two genes.

**Conclusions:**

We verified both up-regulated and down-regulated miRNAs in the tumor samples, indicating various roles of microRNAs during tumor development. Mir-34a functions as a regulator by decreasing the expression of *NOTCH1* and *DLL1*. Our study is the first to identify a correlation between mir-34a and its target genes *NOTCH1* and *DLL1* in endometrial adenocarcinoma.

**Electronic supplementary material:**

The online version of this article (doi:10.1186/s12885-016-2296-z) contains supplementary material, which is available to authorized users.

## Background

Endometrial cancer (EC) is the most frequently diagnosed gynaecological malignancy in the female population of the developed countries. According to the World Cancer Research Fund (WCRF), 320 000 new cases of endometrial cancer were diagnosed worldwide in 2012 [1]. The most common subtype, endometrioid adenocarcinoma (EAC), accounts for approximately 75 % of EC cases [2]. EAC occurs in pre- and post-menopausal women and develops from endometrial hyperplasia. Patients are generally treated with a combination of surgery, chemotherapy and radiotherapy, and in some cases, hormone therapy is applied [3]. The five-year survival rate in patients with early stage of the disease is approximately 80 %, while about 15–20 % develop metastasis [4]. The prognosis is poor for patients with advanced-stage or recurrent endometrial adenocarcinoma due to limited effectiveness of treatment. Understanding the pathogenesis of EAC may provide a basis for development of novel therapeutic strategies.

The discovery of microRNAs (miRNA) has brought new insights into the pathogenesis of various types of diseases including cancer. MiRNAs are a class of non-coding RNA molecules that regulate gene expression post-transcriptionally, usually through incomplete complementary binding to the 3′–untranslated region (3′-UTR) of target mRNA, causing transcript degradation or translation inhibition [5]. So far more than 2500 miRNAs have been discovered in human (miRBase v.20), and the number is still increasing. Since miRNAs may have multiple target genes, it is estimated they may be able to regulate up to 60 % of protein-coding genes, which make miRNAs one of the most important classes of regulators in mammals [6]. The relevance of microRNAs in cancer is related to their regulation of essential cellular processes and pathways, such as cell proliferation, differentiation and apoptosis. The first association of miRNAs and cancer was described by Calin and colleagues in 2002, who found down-regulation of two miRNAs, mir-15 and mir-16 in most patients with chronic lymphocytic leukaemia [7]. Ever since, aberrant miRNA expression levels have been observed in several types of tumors like breast [8], endometrial adenocarcinoma [9] and other solid tumors [10].

We previously identified 138 dysregulated miRNAs in endometrial adenocarcinoma, including mir-34a, which was significantly overexpressed in the cancer samples with a 5-fold change [11]. MiRNA-34a belongs to the family miRNA-34, which also includes mir-34b and mir-34c. Mir-34b and mir-34c are transcribed from a common miRNA gene located on HSA11, while mir-34a is located on HSA1. Mir-34a has been shown to have an aberrant expression in various human tumors [12, 13] and to regulate several genes involved in the Notch signaling pathway [14].

Abnormal Notch signaling has been reported in several cancer types and associated with tumorigenesis [15]. The Notch pathway is one of the basic signaling pathways that regulate tissue development and in addition influences a broad range of events, including proliferation, differentiation and apoptosis in various cell types [16, 17]. In mammals, there are four Notch transmembrane receptors (*NOTCH1-4*), and five transmembrane ligands, which include three Delta-like proteins (*DLL1*, *DLL2*, and *DLL4*) and two Jagged proteins (*JAG1, JAG2*). After activation by ligand binding, the receptor is proteolytically cleaved, which releases an active form of the Notch intracellular domain (NICD) from the plasma membrane. The NICD subsequently translocates to the nucleus and functions as a transcriptional activator to enhance the expression of target genes [18]. It has been observed that aberrant activation of the Notch signaling pathway promotes proliferation in a variety of cancer cell types, including endometrial adenocarcinoma [19, 20].

Previous studies have proven a direct interaction of mir-34a to both *NOTCH1* [21, 22], and *DLL1* [23] using Luciferase activation assays and western blot.

The purpose of the current study is to validate some of the miRNAs that we previously demonstrated to be differentially expressed in EAC [11]. Here we included the two miRNAs that were the most up-regulated in our previous study (mir-182 and mir-183) and one of the downregulated miRNAs (mir-214). Selected were also miRNAs that belong to families with known association to several cancer forms. This includes five miRNAs from the mir-200 family (mir-200a, mir-200b, mir-200c, mir-429 and mir-141), six from the mir-17-92 cluster (mir-18a, mir-18a*, mir-92a-1*, mir-17, mir-17* and mir-20a*), and two from the mir-17 family (mir-106a and mir-106b*). We also choose to include seven miRNAs (mir-370, mir-337-5p, mir-376c, mir-377, mir-1247, mir-758 and mir-300) that are located on the q arm of chromosome 14, which has been found to be aberrant and implicated in many types of cancer [24]. Two additional miRNAs (mir-34a and mir-185) that are involved in many types of cancer [25, 26] were included. We also investigated the possible involvement of mir-34a in the development of EAC by using gain- and loss-of-function experiments to study its regulation of *NOTCH1* and *DLL1*.

In this study it was revealed that several miRNAs were dysregulated in EAC and that expression of *NOTCH1* and *DLL1* mRNA levels were negatively regulated by mir-34a in the Ishikawa and HEK293 cell lines.

## Methods

### Tissues and cell lines

A total of 50 archived formalin-fixed, paraffin-embedded (FFPE) tissue blocks of normal endometrium (20 samples) and endometrial adenocarcinoma (30 samples) were obtained from the University Hospital of Örebro. The normal endometrial samples were collected from patients who had undergone hysterectomy for nonmalignant conditions. Ten of the normal endometrial samples were in the proliferative phase and ten in secretory phase. Patients were staged according to the International Federation of Gynecology and Obstetrics (FIGO) classification system in 1998, and accordingly 10 tumors were classified as stage I, 10 as stage II, and 10 as stage III. Written informed consent for the use of tissues for research was obtained from patients and healthy donors. The study was reviewed and approved by the Regional Ethical Committee Uppsala-Örebro (number 2011/123).

The human endometrial cancer cell line Ishikawa, and the human embryonic kidney 293 (HEK293) cell line were used to study the possible involvement of mir-34a in tumorigenesis, by investigating the relationship between mir-34a and two of its target genes (*NOTCH1* and *DLL1*). The Ishikawa cell line was cultured in Minimum Essential Medium Eagle’s (MEM) supplemented with 5 % fetal Bovine serum, L-Glutamine and 1 % Non Essential Amino Acids. The HEK293 cell line was cultured in Dulbecco’s modified Eagle medium (DMEM) supplemented with 10 % fetal bovine serum, L-Glutamine, 100 IU/100 μg ml − 1 penicillin/streptomycin. The cell lines were grown at 37 °C in an atmosphere of 95 % humidity and 5 % CO_2_.

### MiRNAs included in the study

We used the Pick-&-Mix microRNA PCR panels for validation of the selected miRNAs. The panels included primers for 25 differentially expressed miRNAs (*P <* 0.001), three endogenous control genes, an interplate calibrator and the primer set for detection of a synthetic RNA spike-in (see Additional file 1). All reactions were run in triplicates. The panel included mir-182, mir-183, mir-214, mir-200a, mir-200b, mir-200c, mir-429, mir-141, mir-18a, mir-18a*, mir-92a-1*, mir-17, mir-17*, mir-20a*, mir-106a, mir-106b*, mir-370, mir-337-5p, mir-376c, mir-377, mir-1247, mir-758, mir-300, mir-34a and mir-185.

### RNA extraction

Extraction of RNA from FFPE tissues was performed using a Recover All Total Nucleic Acid Isolation Kit optimized for FFPE samples (Ambion, Foster City, CA, USA) according to the manufacturer’s protocol. Furthermore, total RNA was isolated from the cells using a mirVana miRNA Isolation Kit (Ambion) following the manufacturer’s protocol. The concentration and purity of the RNA were measured using the NanoDrop ND-1000 Spectrophotometer (NanoDrop Technologies, USA).

### Reverse transcription and qPCR for miRNA expression analysis

Reverse transcription was performed using 2 μl RNA in 10 μl reactions using the miRCURY LNA Universal RT miR PCR, polyadenylation and cDNA synthesis kit (Exiqon, Denmark). For quality control synthetic RNA spike-in was added to all total RNA samples prior to labeling and reverse transcription. The cDNA products were diluted 100x with RNase-free water and assayed in 10 μl PCR reactions according to the manufacturer’s protocol; each miRNA assayed by qPCR on the Pick-&-Mix microRNA PCR panels (Exiqon, Denmark). All qPCR reactions were performed in triplicates in a LightCycler 480 real-time PCR system (Roche) in 96 well plates.

### Reverse transcription and qPCR for NOTCH1 and DLL1 mRNA expression analysis

Reverse transcription was performed on 200 ng of total RNA, using the High Capacity RNA-to-cDNA Kit (Applied Biosystems, USA) according to the manufacturer’s protocol. Amplification of *NOTCH1*, *DLL1* and the endogenous control *GAPDH* was performed in 20 μl reactions. Each reaction included 1 μl of 20**✕** TaqMan Gene Expression Assay (*NOTCH1* Hs01062014_m1, *DLL1* Hs00194509_m, *GAPDH* Hs02758991_g, Applied Bio systems), 10 μl of 2**✕** TaqMan Gene Expression Master Mix, 4 μl of cDNA (20 ng) and 5 μl water. All qPCR reactions were performed in triplicates. The reactions were run on an Applied Biosystems 7300 Real Time PCR system with the following thermal cycles: one cycle of 50 °C for 2 min; one cycle of 95 °C for 10 min; 40 cycles with a denaturation step at 95 °C for 15 s and an annealing/extension step at 60 °C for 60 s. The relative expression was calculated using the delta Ct method and the expression of *NOTCH1* and *DLL1* was normalized to *GAPDH*.

### Transfection with a synthetic mir-34a mimic and inhibitor

The Ishikawa and HEK293 cell lines were transfected with mir-34a inhibitor, mir-34 mimic and their respective negative controls by using the Lipofectamine® RNAiMAX Transfection Reagent (Life Technologies) in antibiotic-free Opti-MEM medium (Life Technologies) according to the manufacturer’s protocol at a final concentration of 100 μM. After 24 h, the medium was changed and total RNA was collected 48 h after transfection for further analysis. Transfection efficiency was confirmed with the use of a commercially available kit (Block-iT Alexa Fluor Red Fluorescent Oligo; Life Technologies). All transfections were carried out in duplicates.

### Data pre-processing

The qPCR expression data were imported from the cycler into the GenEx software (MultiD Analyses AB, Göteborg, Sweden). Initially, raw Ct values were adjusted by interplate calibration to compensate for differences between runs. An RNA spike-in control (UniSp6) was used to monitor the efficiency of the RT reactions. Expression of miRNAs was normalized using geometric means of the expression of three stable endogenous control genes, SNORD49A, SNORD38B and hsa-miR-423-5p, which were shown in our previous study to be stably expressed in endometrial tissue [11]. Normalization was performed to the geometric means of the expression of the endogenous control genes, followed by log2 transformation of the normalized Ct values (see Additional file 2).

### Statistical analysis

All qPCR experiments were carried out in triplicates. Statistical analysis of qPCR data was performed using the GenEx software. The two-sided Student’s *t*-test with a stringent *p*-value threshold (*P <* 0.01) was used to determine differences in miRNA and gene expression between normal endometrium and endometrial adenocarcinoma. For comparison, a Mann–Whitney test was also applied (*P <* 0.01). Pearson’s correlation coefficient was used to assess the correlation between the expression levels of mir-34a and its target genes *NOTCH1* and *DLL1* (*P <* 0.01).

## Results

### Dysregulated miRNA in endometrial adenocarcinoma versus normal endometrium

In a previous study we quantified the expression levels of 742 miRNAs in 30 cancer and 20 normal endometrium samples, among these, the commonly down-regulated miRNA mir-34c showed no significant down-regulation in the EAC samples, and mir-101 were up-regulated in the EAC samples compared to normal [11]. A total of 128 miRNAs were up-regulated and 10 were down-regulated in EAC. Hierarchical clustering of the 138 differentially expressed miRNAs showed a clear distinction between normal and cancer tissues with only one exception (one cancer sample clustered among the normal samples). To confirm the dysregulation of those miRNAs, we here validated the expression of 25 selected miRNAs using qPCR in the same tumor and normal samples as in the previous study.

Among the 25 miRNAs that were tested, seven miRNAs were down-regulated and 18 miRNAs were up-regulated compared to normal endometrium (Table 1), which was fully consistent with previous findings.

**Table 1:** List of 25 miRNAs that were differentially expressed in endometrial adenocarcinoma compared to normal endometrium

microRNA	Fold change	*P*-value
mir-183	15.99	3.39E-42
mir-182	13.46	5.11E-43
mir-429	11.40	7.68E-42
mir-200b	8.26	7.77E-39
mir-200a	7.38	4.23E-36
mir-141	4.70	7.02E-29
mir-18a	3.65	5.06E-07
mir-200c	3.40	2.37E-30
mir-18a*	3.03	2.72E-13
mir-106a	2.73	1.31E-18
mir-17	2.68	1.42E-14
mir-34a	2.63	6.12E-15
mir-92a-1*	2.47	1.13E-10
mir-106b*	2.34	5.30E-15
mir-20a*	2.34	8.48E-13
mir-17*	1.99	2.34E-12
mir-185	1.85	9.55E-11
mir-1247	−5.31	2.48E-13
mir-376c	−3.64	6.41E-15
mir-377	−3.34	2.42E-14
mir-214	−2.90	4.90E-12
mir-370	−2.68	2.23E-14
mir-337-5p	−1.94	2.11E-06
mir-300	1.56	3.77E-05
mir-758	−1.61	9.00E-05

### MicroRNA-34a*, NOTCH1* and *DLL1* expression in endometrial adenocarcinoma

Aberrant expression of mir-34a has been observed in several cancers including acute myeloid leukaemia and endometrial adenocarcinoma [11, 27, 28]. To investigate the role of mir-34a in the development of EAC in the present study material, the expression level of mir-34a in human endometrial adenocarcinoma and healthy endometrium was investigated by qPCR. The expression level of mir-34a EAC samples was higher than in the normal samples, which suggests that mir-34a plays a role in development of EAC.

By qPCR we next examined the expression of *NOTCH1* and *DLL1* in a subset comprising 12 tumor tissues (4 at stage I, 4 at stage II and 4 at stage III) and 6 normal samples, where three were in the proliferative phase and three in secretory phase. Our results revealed that mRNA expression of *NOTCH1* and *DLL1* were significantly lower (*P <* 10^−08^) in the tumors than in normal specimens. The expression of *NOTCH1* and *DLL1* in FIGO I-II stages was significantly lower than in FIGO III stage (*P <* 0.01). The relationship between *NOTCH1*, *DLL1* and mir-34a was investigated using Pearson correlation test, which showed a negative correlation between mir-34a and *NOTCH1* (*r =* −0.62, *p =* 0.0056) and *DLL1* (*r =* −0.69, *p =* 0.001) for the sample set as a whole. The correlation prevailed when tested separately in the tumor and normal groups, although with non-significant *p*-values in the normal group.

### Mir-34a inhibitor enhances and mir-34a mimic suppresses *NOTCH1* and *DLL1* mRNA levels

**Fig. 1:**
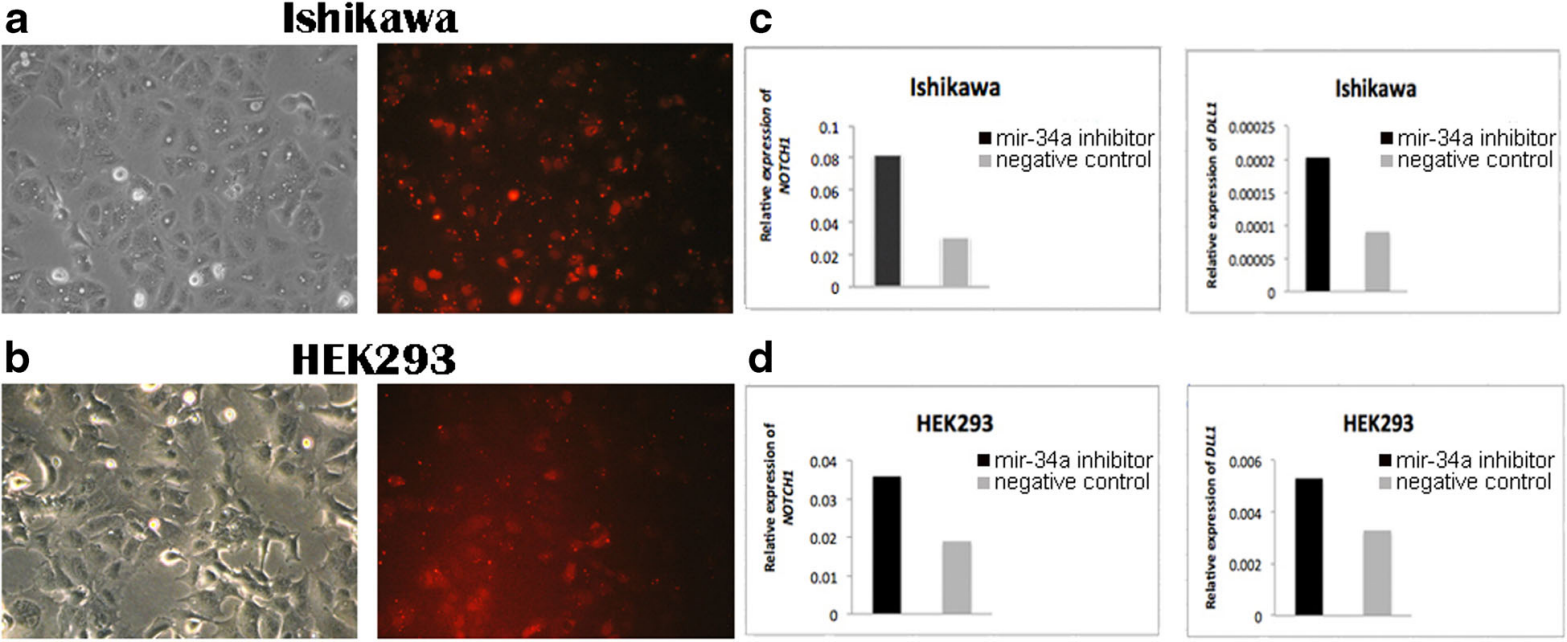
Detection of *NOTCH1* and *DLL1* expression in transfected Ishikawa and HEK293 cell lines. BLOCK-iT Alexa Fluor red fluorescent and mir-34a inhibitor transfection in (**a**) Ishikawa cells and (**b**) HEK293 cells. The photos were taken 24 h after transfection corresponding to morphology of cells (*left*) and BLOCK-iT Alexa Fluor red fluorescent (*right*). **c** The level of *NOTCH1* and *DLL1* expression in Ishikawa cells 48 h after transfection with mir-34a inhibitor compared with the negative control. **d** The level of *NOTCH1* and *DLL1* expression in HEK293 cells 48 h after transfection with mir-34a inhibitor compared with the negative control

**Fig. 2:**
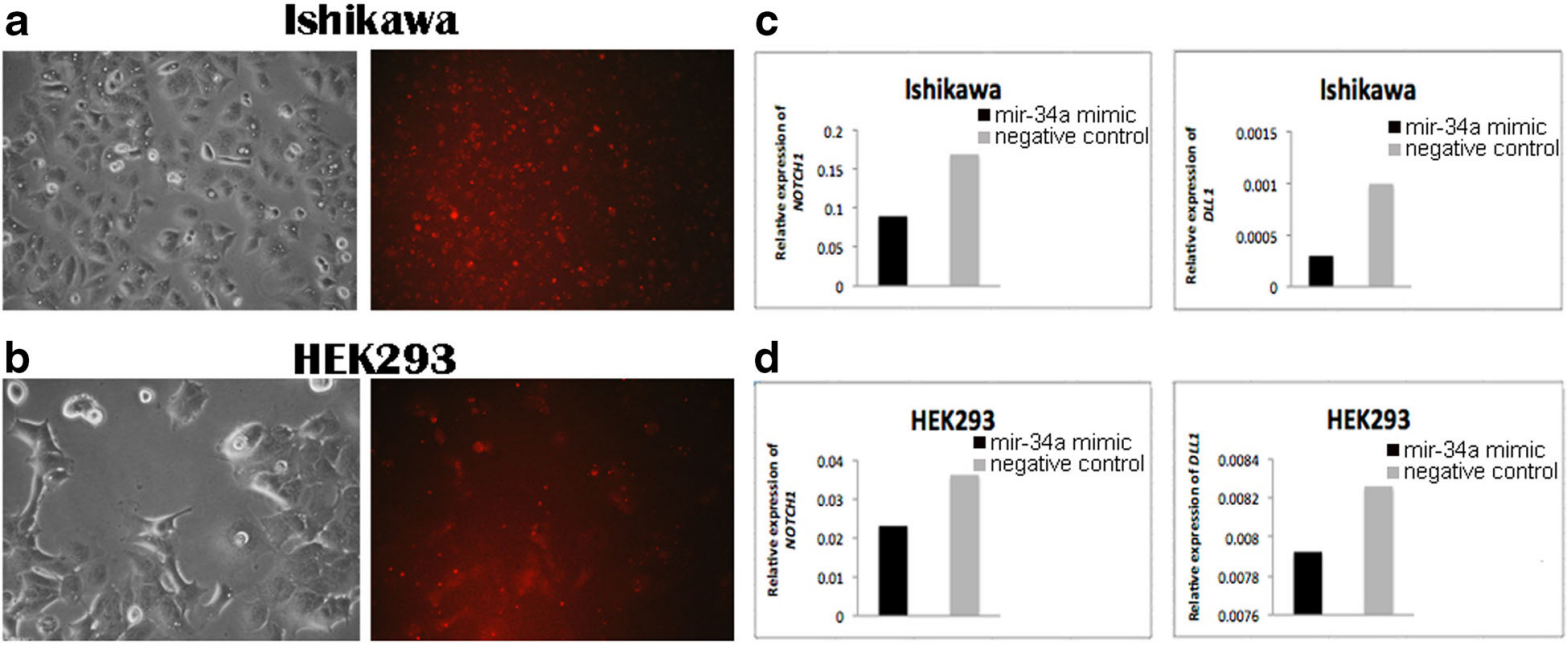
Detection of *NOTCH1* and *DLL1* expression in transfected Ishikawa and HEK293 cell lines. BLOCK-iT Alexa Fluor red fluorescent and mir-34a mimic transfection in (**a**) Ishikawa cells and (**b**) HEK293 cells. The photos were taken 24 h after transfection corresponding to morphology of cells (*left*) and BLOCK-iT Alexa Fluor red fluorescent (*right*). **c** The level of *NOTCH1* and *DLL1* expression in Ishikawa cells 48 h after transfection with mir-34a mimic compared with the negative control. **d** The level of *NOTCH1* and *DLL1* expression in HEK293 cells 48 h after transfection with mir-34a mimic compared with the negative control

To verify whether mir-34a is a regulator of *NOTCH1* and *DLL1* in human endometrial cancer cells, we designed experiments of gain- and loss-of-function of mir-34a in the Ishikawa cell line. To further verify also in another cell type we repeated the experiments in the embryonic kidney cell line HEK293. A mir-34a inhibitor, a mimic, and the corresponding negative controls were transfected into Ishikawa and HEK293 cells and the resulting effects on the levels of *NOTCH1* and *DLL1* expression were monitored by qPCR. The relative expression of *NOTCH1* and *DLL1* was normalized to *GAPDH*. Transfection efficiency was confirmed by using Block-iT Alexa Fluor Red Fluorescent Oligo (Figs. 1a, b and 2a, b).

Gene expression of *NOTCH1* and *DLL1* in the Ishikawa and HEK293 cell lines was significantly increased in the mir-34a inhibitor group compared with the control group (Fig. 1c, d). In addition, the *NOTCH1* and *DLL1* mRNA levels were significantly reduced in both cell lines that were transfected with the mir-34a mimic, compared with those transfected with the mimic negative control (Fig. 2c, d). Both findings confirmed that mir-34a is targeting *NOTCH1* and *DLL1,* and thereby influence their mRNA levels.

## Discussion

Determination of differences in molecular characteristics of cancer and normal tissues is helpful for understanding the complexity in the development of cancer and for developing effective prognostic and diagnostic tools. In a previous study we have used qPCR to identify miRNAs that may contribute to development of endometrial adenocarcinoma. We investigated the expression of 742 miRNAs, and identified 138 to be differentially expressed in EAC compared to normal tissues [11]. Most of the dysregulated miRNAs were significantly up-regulated in EAC, suggesting that miRNAs contribute to tumor progression primarily by repressing the expression of tumor suppressor genes.

The miRNA mir-34c is a known tumor suppressor in other cancer forms, however it is not confirmed in EAC, mir-34c was shown to be low expressed in endometrial cancer cell lines [29], however no studies confirm this in primary tumors, including our material [11]. Even though mir-101 is down-regulated in many cancer forms, the up- or down-regulation of mir-101 is not obvious in EAC, both as it is a part of the TrkB-STAT3 regulatory response, where mir-101 gets up-regulated by increased TrkB levels, and TrkB protein levels is increased in many endometrial carcinoma samples [30]. The stage of the endometrial carcinoma also makes a big difference on the mir-101 level, since it is up-regulated in stage 1 compared to normal, however down-regulated in higher stages compared to normal [31]. In the material used in this study we found an up-regulation of mir-101 (fold change 5,1; *p =* 1,19e-7) in EAC compared to healthy tissue [11]. An important limitation of both this and the previous study [11] was that no tumor adjacent tissues from the endometrial cancer patients were included in the study, thus all differential expressions were based on the comparison of the miRNA-expression in endometrium from healthy donors compared to tumors from EAC patients.

In the present study we validated 25 differentially expressed miRNAs by qPCR in the same samples as in the previous study and found that the expression pattern was consistent with the previous findings. For example, the expression of mir-182 and mir-183 were statistically significantly higher in cancer samples compared to normal endometrium. These two miRNAs regulate the tumor-suppressor gene *FOXO1*, which is known to be down-regulated in EAC. The ability of mir-182 and mir-183 to promote FOXO1 repression may play a key role in the development of EAC by enabling tumors to bypass the cell cycle and apoptosis [32]. In addition, we observed overexpression of the mir-200 family, including mir-200a, mir-200b, mir-200c, mir-141 and mir-429. Gregory and colleagues reported evidence that suggests an essential role for the miR-200 family members in regulation of *ZEB1* and *ZEB2* genes and in the induction of epithelial to mesenchymal transition (EMT) in several types of cancer [33]. Moreover, an inhibition of the miR-200 family using specific anti-miRs resulted in inhibition of cell proliferation in endometrial cancer HEC-1A and Ishikawa cell lines [34].

Park et al. have investigated the expression pattern of mir-200c and its role in cell growth in EAC. They report overexpression of mir-200c in EAC. Moreover, they showed that mir-200c inhibits expression of the tumor suppressor gene *BRD7*. The authors suggested that mir-200c regulates the translocation of *β-catenin* from the cytoplasm to the nucleus via repression of *BRD7*, resulting in up-regulation of its transcriptional target genes, cyclin D1 and c-myc [35].

In the present study eight miRNAs were down-regulated in endometrial adenocarcinoma, of which seven are located in region 14q32. Aberrations in chromosome 14 have been implicated in many types of cancer [24] and miRNAs located in 14q32 have been shown to be under-expressed in gliomas [24], ovarian cancer [36] and gastrointestinal stromal tumors [37], this may represent the largest tumor suppressive miRNA cluster. Our results validate the down-regulation in EAC of mir-214 [11], which regulates expression of *PTEN*, a tumor suppressor gene that produces a protein with lipid and protein phosphatase function, antagonizing the PI3K/Akt pathway by dephosphorylation of phosphoinositides. *PTEN* mutation occurs in approximately 86 % of EAC cases with microsatellite instability [4]. The mutation of *PTEN* is well documented in endometrial hyperplasia, suggesting that it is an early event in tumorigenesis.

Abnormal expression of mir-185 has been observed in several types of cancer including prostate [38], breast [39] and endometrial cancer [11]. One of miR-185 target genes is *AKT1*, which is involved in the PI3K/Akt signaling pathway [40], which is often altered in EAC and involved in the development of the disease.

The mir-17-92 family includes the mir-17-92 cluster and two paralogs, the mir-106a-363 cluster (located on chromosome X) and the mir-106b-25 cluster (located on chromosome 7) [41]. Overexpression of the mir-17-92 cluster in a variety of human cancers [42] indicates that the miRNAs in this cluster may function as oncogenes and promote cancer development by regulating tumor suppressor genes and genes that control cell differentiation or apoptosis. In a tumor engraftment model, Dews and colleagues reported that up-regulation of the cluster by *Myc* in colonocytes increases tumorigenesis by promoting angiogenesis through direct repression of *TSP-1* and *CTGF* by miR-18a and miR-19, respectively [43]. Another type of cancer in which mir-17-92 is overexpressed is neuroblastoma where it is a poor prognostic indicator [44]. Due to the high degree of sequence similarity between mir-17-92 and its paralogs (mir-106a-363 and mir-106b-25), it is not surprising that these two paralogs share the ability to promote tumorigenesis with miR-17-92. Yu et al., demonstrated that mir-93, which is a member of the miR-106b-25 cluster, is able to influence cell proliferation and colony formation of human colon cancer stem cells [45]. Studies of pancreatic cancer have shown that mir-106a has an oncogenic role through promoting cancer cell proliferation and invasion by targeting TIMP-2 [46].

Several studies have reported down-regulation of mir-34a in human tumors, such as ovarian cancer, prostate carcinoma and melanoma [27, 47], and mir-34a has even been implicated as a potential therapeutic target [25]. However recent data show that mir-34a is up-regulated in head and neck squamous cancer compared to normal [48], as we find in this study in EAC. Also elevated mir-34a levels is associated to drug resistance to docetaxel in breast cancer cells [49]. It has been found that mir-34a regulates the expression of *NOTCH1* [47] and *DLL1* [23] in different types of cancer. Notch is considered an oncogene in several cancers, such as T-cell acute lymphoblastic leukemia, as it regulates differentiation and self-renewal in these tissues, however in other cell types such as myeloid malignancies it acts as a tumor suppressor [50]. Thus the cell context is crucial for the actions of Notch signaling, and consequently likely also for mir-34a regulation of *NOTCH1* expression.

We examined the mRNA level of the *NOTCH1* receptor and the *DLL1* ligand in 12 tumor and 6 normal tissues, and found a significant decrease in their expression levels in EAC compared to normal tissues. Additionally, the expression of *NOTCH1* and *DLL1* in FIGO stages I-II was significantly lower than in FIGO III stage (*P <* 0.01), which suggests that these genes participate in tumorigenesis. Interestingly, correlation analysis between mir-34a and clinicopathological features of EAC showed that mir-34a expression in FIGO I-II stages was significantly higher than in FIGO III stage.

To confirm that mir-34a specifically regulates *NOTCH1* and *DLL1*, synthetic mir-34a inhibitor and mimic were transfected into the Ishikawa and HEK293 cell lines. We thereby demonstrated that mir-34a regulates *NOTCH1* and *DLL1* at the mRNA level. As shown in Fig. 2c and d, transfection of cells with the mir-34a inhibitor resulted in an increase of the NOTCH1 and DLL1 mRNA levels compared to their respective negative controls. As expected, transfection with mir-34a mimic resulted in decreased expression of both *NOTCH1* and *DLL1* mRNA levels.

Thus, aberrant expression of *NOTCH1* and *DLL1* in EAC may be caused by aberrant expression of mir-34a, and in conclusion mir-34a targets both *NOTCH1* and *DLL1*.

## Conclusions

In summary, our study confirms that several miRNAs are differentially expressed in endometrial adenocarcinoma. Identification of dysregulated miRNA in EAC can potentially be of great prognostic and diagnostic value and should be considered when trying to understand the complexity of the development of endometrial adenocarcinoma.

Furthermore, our study is the first to identify a correlation between mir-34a and its target genes *NOTCH1* and *DLL1* in endometrial adenocarcinoma. The consistent increase in mir-34a level in EAC, accompanied by a decrease in *NOTCH1* and *DLL1* levels, suggests that mir-34a may serve as a molecular marker of neoplastic transformation in endometrial adenocarcinoma.

### Ethics approval and consent to participate

Written informed consent for the use of tissues for research was obtained from patients and healthy donors. The study was reviewed and approved by the Regional Ethical Committee Uppsala-Örebro (number 2011/123).

### Availability of data and materials

Data is available in the supporting files.

## Additional files

Additional file 1: Pick-&-Mix microRNA PCR panel list. (XLS 30 kb)

Additional file 2: Normalized expression values. (XLS 71 kb)

## Abbreviations

EC: endometrial cancer
EAC: endometrioid adenocarcinoma
miRNA: MicroRNA
3‘-UTR: 3‘ –untranslated region
FFPE: formalin-fixed paraffin-embedded tissue
FIGO: international Federation of Gynecology and Obstetrics
HEK293: human embryonic kidney 293
qPCR: Real-time Quantitative PCR

## References

[1] Ferlay J, Soerjomataram I, Ervik M, Dikshit R, Eser S, Mathers C, Rebelo M, Parkin DM, Forman D, Bray F: GLOBOCAN 2012 v1.0, Cancer Incidence and Mortality Worldwide: IARC CancerBase No. 11[Internet]. Lyon, France: International Agency for Research on Cancer; 2013. Available from: http//globocan.iarc.fr, accessed on 13/12/2013.

[2] Emons G, Fleckenstein G, Hinney B, Huschmand A, Heyl W (2000). Hormonal interactions in endometrial cancer. Endocr Relat Cancer.

[3] Cavanagh D, Fiorica JV, Hoffman MS, Durfee J, Nicosia SV (1999). Adenocarcinoma of the endometrium: an institutional review. Cancer Control.

[4] Amant F, Moerman P, Neven P, Timmerman D, Van Limbergen E, Vergote I (2005). Endometrial cancer. Lancet.

[5] Lee Y, Jeon K, Lee J, Kim S, Kim V (2002). MicroRNA maturation: stepwise processing and subcellular localization. EMBO J.

[6] Friedman RC, Farh KKH, Burge CB, Bartel DP (2009). Most mammalian mRNAs are conserved targets of microRNAs. Genome Res.

[7] Calin GA, Dumitru CD, Shimizu M, Bichi R, Zupo S, Noch E, Aldler H, Rattan S, Keating M, Rai K (2002). Frequent deletions and down-regulation of micro-RNA genes miR15 and miR16 at 13q14 in chronic lymphocytic leukemia. Proc Natl Acad Sci.

[8] Iorio MV, Ferracin M, Liu CG, Veronese A, Spizzo R, Sabbioni S, Magri E, Pedriali M, Fabbri M, Campiglio M (2005). MicroRNA gene expression deregulation in human breast cancer. Cancer Res.

[9] Devor EJ, Goodheart MJ, Leslie KK (2011). Toward a microRNA signature of endometrial cancer. Proceedings in Obstetrics and Gynecology.

[10] Molnar V, Tamasi V, Bakos B, Wiener Z, Falus A (2008). Changes in miRNA expression in solid tumors: an miRNA profiling in melanomas. Semin Cancer Biol.

[11] Jurcevic S, Olsson B, Klinga Levan K (2014). MicroRNA expression in human endometrial adenocarcinoma. Cancer Cell Int.

[12] Zhang D, Zhou J, Dong M (2014). Dysregulation of microRNA-34a expression in colorectal cancer inhibits the phosphorylation of FAK via VEGF. Dig Dis Sci.

[13] Andrew AS, Marsit CJ, Schned AR, Seigne JD, Kelsey KT, Moore JH, et al. Expression of tumor suppressive microRNA-34a is associated with a reduced risk of bladder cancer recurrence. Int J Cancer. 2014. 10.1002/ijc.29413 PMC4485975 25556547

[14] Griffiths-Jones S, Saini HK, van Dongen S, Enright AJ (2008). miRBase: tools for microRNA genomics. Nucleic Acids Res.

[15] Axelson H (2004). Notch signaling and cancer: emerging complexity. Semin Cancer Biol.

[16] Bigas A, Robert-Moreno A, Espinosa L (2010). The Notch pathway in the developing hematopoietic system. Int J Dev Biol.

[17] Bolos V, Grego-Bessa J, de la Pompa JL (2007). Notch signaling in development and cancer. Endocr Rev.

[18] Artavanis-Tsakonas S, Rand MD, Lake RJ (1999). Notch signaling: cell fate control and signal integration in development. Science.

[19] Wei Y, Zhang Z, Liao H, Wu L, Wu X, Zhou D, Xi X, Zhu Y, Feng Y (2012). Nuclear estrogen receptor-mediated Notch signaling and GPR30-mediated PI3K/AKT signaling in the regulation of endometrial cancer cell proliferation. Oncol Rep.

[20] Rose SL (2009). Notch signaling pathway in ovarian cancer. International Journal of Gynecological Cancer.

[21] Li Y, Guessous F, Zhang Y, Dipierro C, Kefas B, Johnson E, Marcinkiewicz L, Jiang J, Yang Y, Schmittgen TD (2009). MicroRNA-34a inhibits glioblastoma growth by targeting multiple oncogenes. Cancer Res.

[22] Li WB, Ma MW, Dong LJ, Wang F, Chen LX, Li XR (2011). MicroRNA-34a targets notch1 and inhibits cell proliferation in glioblastoma multiforme. Cancer Biol Ther.

[23] Pang RT, Leung CO, Lee CL, Lam KK, Ye TM, Chiu PC, Yeung WS (2013). MicroRNA-34a is a tumor suppressor in choriocarcinoma via regulation of Delta-like1. BMC Cancer.

[24] Lavon I, Zrihan D, Granit A, Einstein O, Fainstein N, Cohen MA, Cohen MA, Zelikovitch B, Shoshan Y, Spektor S (2010). Gliomas display a microRNA expression profile reminiscent of neural precursor cells. Neuro Oncol.

[25] Li XJ, Ren ZJ, Tang JH (2014). MicroRNA-34a: a potential therapeutic target in human cancer. Cell Death Dis.

[26] Lu ZJ, Lu LG, Tao KZ, Chen DF, Xia Q, Weng JJ, Zhu F, Wang XP, Zheng P (2014). MicroRNA-185 suppresses growth and invasion of colon cancer cells through inhibition of the hypoxiainducible factor-2alpha pathway in vitro and in vivo. Mol Med Rep.

[27] Lodygin D, Tarasov V, Epanchintsev A, Berking C, Knyazeva T, Korner H, Knyazev P, Diebold J, Hermeking H (2008). Inactivation of miR-34a by aberrant CpG methylation in multiple types of cancer. Cell Cycle.

[28] Isken F, Steffen B, Merk S, Dugas M, Markus B, Tidow N, Zuhlsdorf M, Illmer T, Thiede C, Berdel WE (2008). Identification of acute myeloid leukaemia associated microRNA expression patterns. Br J Haematol.

[29] Jiang L, Meng W, Zeng J, Hu H, Lu L (2013). MiR-34c oligonucleotide enhances chemosensitivity of Ishikawa cell to cisplatin by inducing apoptosis. Cell Biol Int.

[30] Bao W, Wang HH, Tian FJ, He XY, Qiu MT, Wang JY, Zhang HJ, Wang LH, Wan XP (2013). A TrkB-STAT3-miR-204-5p regulatory circuitry controls proliferation and invasion of endometrial carcinoma cells. Mol Cancer.

[31] Torres A, Torres K, Pesci A, Ceccaroni M, Paszkowski T, Cassandrini P, Zamboni G, Maciejewski R (2013). Diagnostic and prognostic significance of miRNA signatures in tissues and plasma of endometrioid endometrial carcinoma patients. Int J Cancer.

[32] Myatt SS, Wang J, Monteiro LJ, Christian M, Ho KK, Fusi L, Dina RE, Brosens JJ, Ghaem-Maghami S, Lam EWF (2010). Definition of microRNAs that repress expression of the tumor suppressor gene FOXO1 in endometrial cancer. Cancer Res.

[33] Gregory PA, Bert AG, Paterson EL, Barry SC, Tsykin A, Farshid G, Vadas MA, Khew-Goodall Y, Goodall GJ (2008). The miR-200 family and miR-205 regulate epithelial to mesenchymal transition by targeting ZEB1 and SIP1. Nat Cell Biol.

[34] Lee JW, Park YA, Choi JJ, Lee YY, Kim CJ, Choi C, Kim TJ, Lee NW, Kim BG, Bae DS (2011). The expression of the miRNA-200 family in endometrial endometrioid carcinoma. Gynecol Oncol.

[35] Park YA, Lee JW, Choi JJ, Jeon HK, Cho Y, Choi C, Kim TJ, Lee NW, Kim BG, Bae DS (2012). The interactions between MicroRNA-200c and BRD7 in endometrial carcinoma. Gynecol Oncol.

[36] Zhang L, Volinia S, Bonome T, Calin GA, Greshock J, Yang N, Liu CG, Giannakakis A, Alexiou P, Hasegawa K (2008). Genomic and epigenetic alterations deregulate microRNA expression in human epithelial ovarian cancer. Proc Natl Acad Sci U S A.

[37] Haller F, von Heydebreck A, Zhang JD, Gunawan B, Langer C, Ramadori G, Wiemann S, Sahin O (2010). Localization- and mutation-dependent microRNA (miRNA) expression signatures in gastrointestinal stromal tumours (GISTs), with a cluster of co-expressed miRNAs located at 14q32.31.. J Pathol.

[38] Qu F, Cui X, Hong Y, Wang J, Li Y, Chen L, Liu Y, Gao Y, Xu D, Wang Q (2013). MicroRNA-185 suppresses proliferation, invasion, migration, and tumorigenicity of human prostate cancer cells through targeting androgen receptor. Mol Cell Biochem.

[39] Fu P, Du F, Yao M, Lv K, Liu Y (2014). MicroRNA-185 inhibits proliferation by targeting c-Met in human breast cancer cells. Exp Ther Med.

[40] Datta SR, Brunet A, Greenberg ME (1999). Cellular survival: a play in three Akts. Genes Dev.

[41] Tanzer A, Stadler PF (2004). Molecular evolution of a microRNA cluster. J Mol Biol.

[42] Mogilyansky E, Rigoutsos I (2013). The miR-17/92 cluster: a comprehensive update on its genomics, genetics, functions and increasingly important and numerous roles in health and disease. Cell Death Differ.

[43] Dews M, Homayouni A, Yu D, Murphy D, Sevignani C, Wentzel E, Furth EE, Lee WM, Enders GH, Mendell JT (2006). Augmentation of tumor angiogenesis by a Myc-activated microRNA cluster. Nat Genet.

[44] Mestdagh P, Bostrom AK, Impens F, Fredlund E, Van Peer G, De Antonellis P, von Stedingk K, Ghesquiere B, Schulte S, Dews M (2010). The miR-17-92 microRNA cluster regulates multiple components of the TGF-beta pathway in neuroblastoma. Mol Cell.

[45] Yu XF, Zou J, Bao ZJ, Dong J (2011). miR-93 suppresses proliferation and colony formation of human colon cancer stem cells. World J Gastroenterol.

[46] Li P, Xu Q, Zhang D, Li X, Han L, Lei J, Duan W, Ma Q, Wu Z, Wang Z (2014). Upregulated miR-106a plays an oncogenic role in pancreatic cancer. FEBS Lett.

[47] Misso G, Di Martino MT, De Rosa G, Farooqi AA, Lombardi A, Campani V, Zarone MR, Gulla A, Tagliaferri P, Tassone P (2014). Mir-34: a new weapon against cancer?. Mol Ther Nucleic Acids.

[48] Kalfert D, Pesta M, Kulda V, Topolcan O, Ryska A, Celakovsky P, Laco J, Ludvikova M (2015). MicroRNA profile in site-specific head and neck squamous cell cancer. Anticancer Res.

[49] Kastl L, Brown I, Schofield AC (2012). miRNA-34a is associated with docetaxel resistance in human breast cancer cells. Breast Cancer Res Treat.

[50] Lobry C, Oh P, Mansour MR, Look AT, Aifantis I (2014). Notch signaling: switching an oncogene to a tumor suppressor. Blood.

